# An experimental comparison of multiple-choice and short-answer questions on a high-stakes test for medical students

**DOI:** 10.1007/s10459-023-10266-3

**Published:** 2023-09-04

**Authors:** Janet Mee, Ravi Pandian, Justin Wolczynski, Amy Morales, Miguel Paniagua, Polina Harik, Peter Baldwin, Brian E. Clauser

**Affiliations:** 1 NBME, Philadelphia, USA; 2 American College of Physicians, Philadelphia, USA

**Keywords:** Multiple choice, Short answer, Constructed response, Item performance

## Abstract

Recent advances in automated scoring technology have made it practical to replace multiple-choice questions (MCQs) with short-answer questions (SAQs) in large-scale, high-stakes assessments. However, most previous research comparing these formats has used small examinee samples testing under low-stakes conditions. Additionally, previous studies have not reported on the time required to respond to the two item types. This study compares the difficulty, discrimination, and time requirements for the two formats when examinees responded as part of a large-scale, high-stakes assessment. Seventy-one MCQs were converted to SAQs. These matched items were randomly assigned to examinees completing a high-stakes assessment of internal medicine. No examinee saw the same item in both formats. Items administered in the SAQ format were generally more difficult than items in the MCQ format. The discrimination index for SAQs was modestly higher than that for MCQs and response times were substantially higher for SAQs. These results support the interchangeability of MCQs and SAQs. When it is important that the examinee generate the response rather than selecting it, SAQs may be preferred. The results relating to difficulty and discrimination reported in this paper are consistent with those of previous studies. The results on the relative time requirements for the two formats suggest that with a fixed testing time fewer SAQs can be administered, this limitation more than makes up for the higher discrimination that has been reported for SAQs. We additionally examine the extent to which increased difficulty may directly impact the discrimination of SAQs.

## Introduction

The use of multiple-choice questions (MCQs) for assessment in medical education has been controversial since the item type first came into wide use. When the National Board of Medical Examiners (NBME) introduced MCQs to its examinations in the 1950s, some state medical boards were so opposed to the item type that they dropped their recognition of the examinations for licensure (Hubbard & Levit, 1985, p. 28). Since then, numerous studies have compared MCQs and constructed response items, and authors continue to argue strenuously for or against using MCQs (e.g., Hift, 2014; Sam et al., 2016; Schuwirth & van der Vlueten, 2004). Researchers have raised concerns about the extent to which the two formats assess different constructs. The option list, which is a defining feature of the MCQ format, has raised concerns both because it creates the possibility of guessing and because it changes the challenge from recall to recognition of the correct answer. To minimize the impact of guessing, some test developers have used a format referred to as *extended matching* in which the option list is substantially longer than with a typical MCQ. Empirical evaluation of the format has shown that presenting items with longer option lists makes them more difficult—which may be evidence of reduced success based on guessing. This item format also requires more testing time than the typical MCQ format (Swanson et al., 2006). However, the extended matching format still allows for responses based on recognition rather than recall, which some health sciences educators view as problematic. In response to these concerns, particular attention has been given to short-answer questions (SAQs). With this format, the text of a test item may be identical to that of an MCQ, but rather than selecting an option, the examinee responds using free text.

Interest and controversy aside, the differences were of little practical concern for large-scale testing because the higher cost and lower accuracy of scoring SAQs made them impractical. Recent advances in natural language processing (NLP) and machine learning have made accurate computer scoring of SAQs achievable (e.g., Sam et al., 2019; Yamamoto et al., 2017). The work of Suen et al. (2023) is of particular relevance in the context of the present study. These researchers scored the responses used in the present study using an AI driven system based on a large language model. They reported an average level of agreement of 0.98 between human and machine scores when the system was trained on a modest to moderate sample of examinee responses. Given these advances in machine scoring, the issue of the relative performance of the two formats is no longer a matter of academic interest but one of practical concern.

There are numerous previous studies that report results that are relevant to the question of how performance differs across these two formats. These studies generally support expectations that SAQs are more difficult than MCQs and that the two formats have similar levels of reliability (or discrimination). These results may be sufficient to guide health-professions educators in selecting a format for classroom assessment. The results are not appropriate for making decisions about the large-scale and high-stakes applications that are made possible by AI scoring. In these contexts, it is important to quantify the tradeoffs associated with each format and in this regard the previously published studies have significant limitations. Perhaps the most significant problem with previous research is that experimental designs with random assignment are largely absent. Numerous studies use quasi-experimental designs in which the same examinees respond to the same items in both formats (e.g., Sam et al., 2019); such designs are at best limited. We know of no previous study that used random assignment with matched MCQs and SAQs. A second important limitation of the previous research is that (to our knowledge) no previous study has provided empirical results about the amount of time required to respond to the two formats. One exception is a study by Sam et al. (2019) in which they report that responding to 50 SAQs required an average of 82 min and responding to 50 matched MCQs required 24 min. It is, however, essentially impossible to compare these measures because when the examinees were responding to the MCQs, they were reading the items for the second time. Since testing time is often a limiting factor in developing high-stakes tests, understanding this aspect of the tradeoff between formats is essential. In addition to these two important limitations of the previous literature, we note that previous results have typically (or exclusively) been based on small examinee samples collected under low-stakes conditions. (A more detailed discussion of the limitations of the previous research is provided in our literature review.)

To fill these important gaps in the literature, in this study, the items were administered as part of the NBME Subject Examination program. Among other things, this meant that examinees were motivated to perform as well as possible and that the associated test material was professionally developed and edited before use. Matched MCQs and SAQs were randomly assigned to examinees with no examinee seeing the same item content in both formats. Additionally, because this study was computer-administered, specific timing data for each item was collected.

In what follows, we summarize previous research comparing MCQs to constructed response items, giving particular attention to studies comparing MCQs and SAQs. We then present the results of our study comparing the performance of content-matched MCQs and SAQs. Finally, we discuss the practical implications of the results for assessment in health science education.

## Literature review

Numerous previous papers have reported on comparative studies of multiple-choice and short-answer questions. Schuwirth and van der Vlueten (2004) summarize some of the earlier research making this comparison. They acknowledge that MCQs provide a level of cuing and that there may be specific competencies for which one format might be preferable. However, they conclude that “the response format has less influence on what is being measured than we may be inclined to think” (p. 977). In contrast, some authors seem to take it as given that MCQs are flawed. For example, Sam et al. (2016) conclude that replacing MCQs with SAQs will improve assessment because it “will test nascent physician ability rather than ability to pass exams” (p. 1). More specifically, they comment that

Students who practise large numbers of past questions can become adept at choosing the correct option from a list without an in-depth understanding of the topic. While practising exam questions can increase knowledge, the use of cues to exclude distractors is an important skill in exam technique with [single best answer items] SBAs. This technique improves performance in the assessment, but does not enhance the student’s ability to make a diagnosis in clinical situations. (p. 3)

### Research on item performance

Interpretations aside, some results seem to generalize across studies. Most notably, numerous studies have found that scores tend to be higher for MCQs than for SAQs. Sam et al. (2016) used a design in which the same 15 item stems were presented to examinees in both MCQ and SAQ format. The items were presented first as SAQs and then presented as MCQs. Examinees performed uniformly higher when seeing the items in the MCQ format. In a more extensive follow-up study, Sam et al. (2018) used 60 items in each format and presented the items in a counter-balanced design, with one group responding to the SAQs first and the other responding to the MCQs first. Examinees had higher scores when the items were presented as MCQs, but the difference in scores was substantially reduced when the items were presented in the MCQ format first. Sam et al. (2019) provided an even larger follow-up that specifically addressed whether the performance difference could be accounted for by the fact that with MCQs, random guessing will result in a score of approximately 20% correct (for items with five options). The results substantially—and significantly—exceeded that baseline.

These results reported by Sam and colleagues (Sam et al., 2016, 2018, 2019) are in general agreement with previous literature. An early study within the scope of medical education by Newble et al. (1979) described two experiments. In the first, items were administered to examinees first as SAQs and then as MCQs. In the second study, examinees responded to items in both formats, but the item stems were not identical across formats. Again, SAQs were reported to be more difficult than MCQs. Schuwirth et al. (1996) report the results of another study that used matched test content; their results show modest but stable differences in difficulty, with MCQs again being easier than SAQs. Additional studies that report results consistent with this general trend include Norman et al. (1987) and Heemskerk et al. (2008).

In addition to comparing the difficulty of items in the two formats, several studies report on measures of reliability or discrimination (item-test correlations). Studies comparing MCQs to complex constructed response items such as essays or simulations routinely show the MCQs to be more reliable per hour of testing time (e.g., Clauser et al., 2002; Wainer & Thissen, 1993). Reported results for SAQs have been quite different. Schuwirth et al. (1996) compared SAQs and MCQs and reported alpha coefficients of 0.88 and 0.87 for the two formats, respectively. Sam et al. (2018) reported on the reliability of tests comprising matched SAQs and MCQs. Because of the counterbalanced design (described previously), two examinee samples completed the tests in each format. Coefficient alpha for the MCQs was 0.84 and 0.85; for the SAQs, the equivalent values were 0.91 for both samples. In a follow-up study, Sam et al. (2019) reported reliabilities of 0.69 and 0.73 for MCQs and SAQs, respectively.

In addition to difficulty and discrimination, some attention has been given to the time required to respond to different item formats. Hift (2014) notes that previous studies have shown that open-ended items require more time than MCQs to achieve equivalent levels of reliability. He reports that open-ended tests require 4 to 40 times as long to administer. This estimate refers to more complex constructed response types, such as essay questions. More information is needed regarding the relative time requirements for MCQs and SAQs. Sam et al. (2018) administered 60-item tests in MCQ and SAQ formats. Examinees were given 90 min to respond to the SAQs and 60 min to respond to the MCQs. This reflects an expectation that SAQs are more time intensive, but empirical results on the amount of time used to respond to the two formats were not provided. Sam et al. (2019) administered a 50-item test in both MCQ and SAQ formats. Two hours were allowed for the SAQ responses, and 1 hour was allocated for the MCQs. The authors report that the SAQs required an average of 82 min and the MCQs 24 min; however, when the examinees were responding to the MCQs, they were reading and responding to the items for the second time, so it is impossible to compare the measures directly.

## Limitations of previous research

Although numerous researchers have reported on comparative studies of SAQs and MCQs, evidence for drawing clear and generalizable conclusions is limited. Much of this research was based on small examinee samples (e.g., Heemskerk et al., 2008; Newble et al., 1979; Norman et al., 1987). Moreover, interpreting these results is difficult because many studies use a design in which items with the same stem are presented consecutively in different formats (typically with the SAQ preceding the MCQ). This approach requires the very strong assumption that no benefit is associated with seeing the same item for a second time. This assumption seems problematic given the extensive literature indicating that answer changes associated with item review lead to net increases in performance (e.g., Bridgeman, 2012; Ouyang et al., 2019). Additionally, the studied items were typically developed by the researchers. This raises questions about the extent to which the performance differences resulted from flawed item writing. Items professionally developed for high-stakes tests—which undergo quality control procedures, careful editing, and pretesting—are less likely to have flaws that allow test-wise examinees to eliminate options based on construct-irrelevant characteristics. Similarly, the responses that form the basis for many—if not all—of these studies were made under low-stakes conditions in which the examinees knew that their responses were part of a research study (Heemskerk et al., 2008; Newble et al., 1979; Norman et al., 1987; Sam et al., 2016, 2018, 2019). Although it is impossible to know how that condition may have impacted the results, it is reasonable to question whether the results gathered under low-stakes conditions generalize to high-stakes administrations. Finally, we found no previous studies that provided interpretable results relating to the difference in testing time required to complete items in the two formats. Some studies reported on the amount of time provided, but the time allocations were not empirically determined, and typically, no evidence was provided about how much of the allocated time was used. The one study that reported on the amount of time used in responding to each format (Sam et al., 2019) failed to account for the fact that when examinees were responding to the MCQs they were reading and responding to the items for the second time. Finally, but perhaps most importantly, we found no previous study in which matched SAQs and MCQs were delivered through random assignment. Either all examinees responded to matched items in both formats or unmatched items were used. These design features allow for confounding factors which could impact the results.

In response to the limitations described in the previous paragraph, the present study conforms to an experimental design and is based on large samples of examinees who responded to the test material as part of a high-stakes administration. The MCQ form of the items was professionally developed for use on USMLE. An SAQ version of each item was created for this study; the stems were unchanged across formats. Finally, the tests were administered by computer, and the time spent on each item was recorded for each examinee.

## Method

The purpose of the study was to evaluate the relative performance of MCQs and SAQs. We were specifically interested in three aspects of item performance: difficulty, discrimination, and response time. The SAQs in this study have sometimes been referred to as *very short answer questions*—responses are a word or a phrase with 60 or fewer characters.

### Materials

The initial sample of studied items comprise 100 MCQs that had previously been used operationally on the USMLE Step 2 CK examination and were planned for future use in the NBME Internal Medicine Subject Exam. In addition to the requirement that the studied items test content appropriate for the Internal Medicine Subject Exam, inclusion in the study was limited to single-best-answer items that met specific statistical criteria based on previous use in the Step 2 CK examination. All selected questions related to one of the following areas: *Laboratory and Diagnostic Studies, Diagnosis, Pharmacotherapy, Clinical Interventions,* and *Mixed Management*. Each MCQ was then used to produce a matched SAQ by removing the option list and appropriately adjusting the lead-in. For example, “which of the following is the most likely diagnosis” would be changed to “what is the most likely diagnosis.” No changes were made to the item stem. The changes to the lead-ins were made by editorial staff and reviewed by a content expert.

### Data collection

Data were collected during operational administration of the NBME Internal Medicine Subject Exam. This exam is typically administered at the end of the internal medicine clerkship. Conditions for administration are standardized, and test scores typically contribute to examinees’ clerkship grades or pass/fail decisions. Data collection occurred over 12 months, from August 2018 to July 2019. During this period, each examinee completing the NBME Internal Medicine Subject Exam responded to four study items: two in the MCQ format and two in the SAQ format. A single examinee never saw the same item in both formats. The result was that a random sample of examinees responded to each of the 100 items in each format.

### Scoring

The MCQs were scored in the usual manner by identifying whether the examinee selected the keyed response and the SAQs were scored by content experts using a scoring rubric. A group of three physicians reviewed and discussed a sample of examinee responses for each SAQ item. The first task posed to the group was to decide if the items were “fair” when presented in SAQ format. After considering the responses the group identified items that were ambiguous as presented and should not be scored. This sort of ambiguity arose because the option set for the MCQs acted as a prompt regarding the nature of the response that was expected. For example, the MCQ form of an item might ask “Which of the following is the best next step in diagnosis” and then list five lab tests. When converted to the SAQ format the lead-in would ask, “What is the best next step in diagnosis?” The physician group believed that such ambiguity was unfair and that it additionally made it essentially impossible to develop a suitable scoring rubric, because the range of potentially defensible responses could become extensive. Although these decisions were based on the physicians’ professional judgement, they were guided by review of the actual examinee responses. After eliminating these ambiguous items, the physicians developed rubrics for each of the remaining items. Four individuals (two nurse practitioners and two physician assistants) then independently applied the rubrics to the examinee responses for each item. If all four independent judgments were the same (correct or incorrect), that unanimous judgment was accepted as the score for that response. The original group of three physicians reviewed any items for which the four raters did not have unanimous agreement and made final scoring decisions for those responses. This consensus-based approach was used to minimize the impact of individual rater errors. Because disagreements between the four raters (nurse practitioners and physician’s assistants) did not impact the final score for an item, it is not possible to quantify the accuracy of the scoring process as a function of rater agreement. Nonetheless, as a general metric of the consistency of the judgments, we report the proportion of unique responses for which two, three, or all four judges agreed. We also calculated the proportion of agreement across responses for all pairs of judges.

### Analysis

Again, item difficulty, item discrimination, and item-level examinee response time were of primary interest in this study. Because the items were randomly assigned to examinees and the sample size per item was reasonably large (the average number of examinees per SAQ was 176 with a standard deviation of 12.6), we estimated item difficulty as a p-value representing the proportion of the random sample correctly answering each item.

Estimating discrimination for each item was more complex because the study items were embedded in multiple operational test forms used during the study period. To account for this, we estimated the discrimination index as the point biserial correlation between each examinee’s 0/1 score on the studied item and the item-response-theory-based proficiency estimate for the scored items on the operational test. Using this proficiency estimate placed all the total-test scores on the same scale regardless of whichever test form an examinee completed. We note that these proficiency estimates were based on the one-parameter logistic model and so the number correct score from any given test form is a sufficient statistic for the item-response-theory-based proficiency. Because of this characteristic, the resulting estimate of discrimination will be consistent with the classical test theory measure of discrimination that would have been produced if all the examinees had responded to the same items.

Finally, because the items were computer-administered, we recorded the time each examinee used to respond to each item. Response time was measured from the second the examinee opened the item until the second they moved to the next item. If an examinee returned to an item, the additional time was included.

In evaluating the potential differences in item performance across formats, it is of interest to compare the distributions of the identified metrics. A mean difference in difficulty, discrimination, or time intensity might arise because each item has become uniformly more difficult, discriminating, or time consuming, respectively, across formats. Such a difference might also arise because a small number of items have become much more difficult (or discriminating or time consuming) while others have been unchanged or have even become modestly easier. One straightforward way to evaluate differences in the distributions across formats is by producing scatter plots and correlations. To the extent that the plotted values adhere closely to the diagonal that moves from the lower left to the upper right (and the correlations approach 1.00) the change in difficulty, discrimination, or time intensity will be relatively uniform. Failure to demonstrate this pattern will be evidence that the specifics of the (unchanged) stem interact with the format to impact performance. To this end, we provide scatter plots and correlations for each of the three statistics: p-value, point-biserial, and mean response time.

Although previous studies have generally reported that SAQs are more difficult than MCQs, it is not uncommon that a subsample of items are more difficult in MCQ format. This phenomenon has been referred to as *negative cueing* (e.g., Sam et al., 2019; Schuwirth et al., 1996). Although previous authors have hypothesized that this performance pattern is associated with items that include a very attractive distractor, they have not empirically evaluated the hypothesis. To explore this phenomenon, we performed a secondary analysis designed to evaluate the extent to which items that are more difficult in MCQ format tend to have a single distractor that is selected by a disproportionate proportion of examinees. A graph was produced plotting the item difficulty (p-value for the keyed response) against the proportion of examinees selecting the most attractive distractor (p-value for the most frequently selected distractor), using different markers for the items that were more difficult in MCQ and SAQ format. If visual examination shows that items that are more difficult in MCQ format also have a single frequently selected distractor the result would support the explanation provided by previous researchers—if not, that explanation may be suspect. To further quantify this effect, we also implemented a t-test.

## Results

As previously described, a group of three physicians reviewed a sample of responses for each item while developing the scoring rubrics. The first question posed to the physicians was whether the item should be considered “fair” when presented as an SAQ. The physicians concluded that 29 items were too ambiguous (when presented in SAQ form) to be scored objectively. At this stage these 29 of the 100 studied items were set aside.

**Fig. 1:**
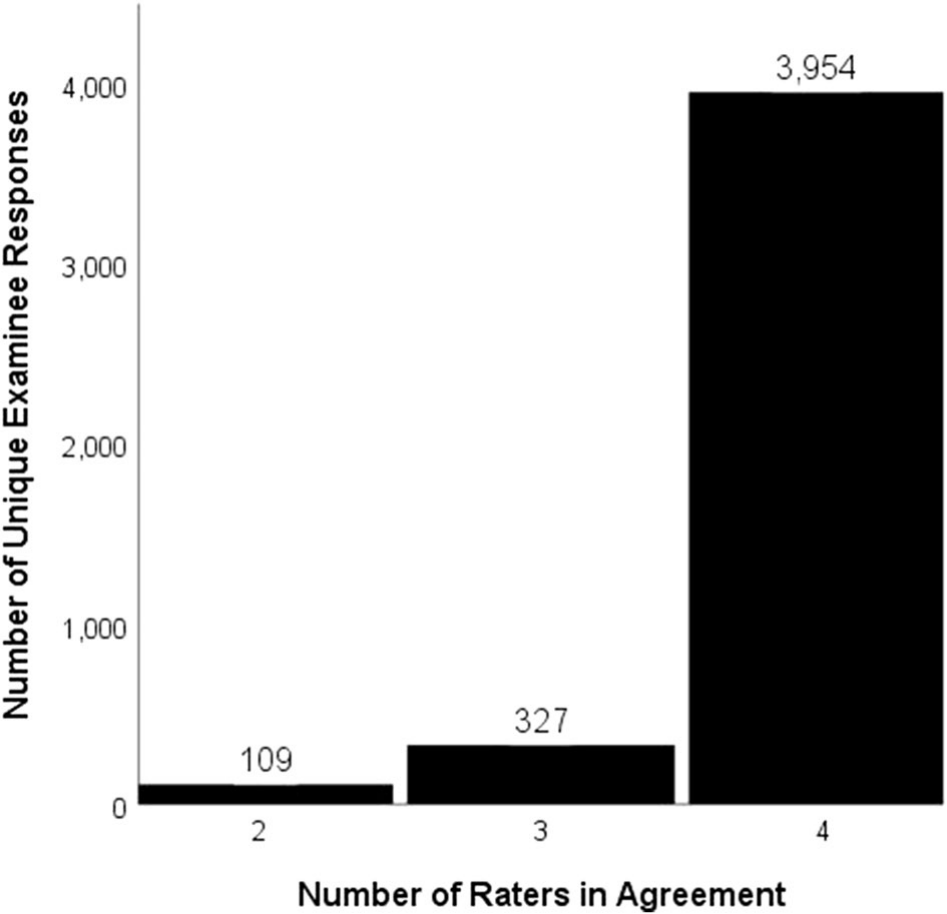
Number of raters agreeing when scoring short-answer question responses

The content experts then developed scoring rubrics for each of the remaining 71 SAQs. Again, these items were independently scored by four judges (nurse practitioners and physician assistants) trained to use the rubrics developed by the physicians. The level of agreement between the judges is presented in Fig. 1. As the figure shows, all four judges agreed on 90% of the *unique* examinee responses (far more than 90% of all responses). The proportion of agreement for the six possible pairs of judges was 0.98 across all responses and 0.95 for the unique responses.

### Difficulty

**Fig. 2:**
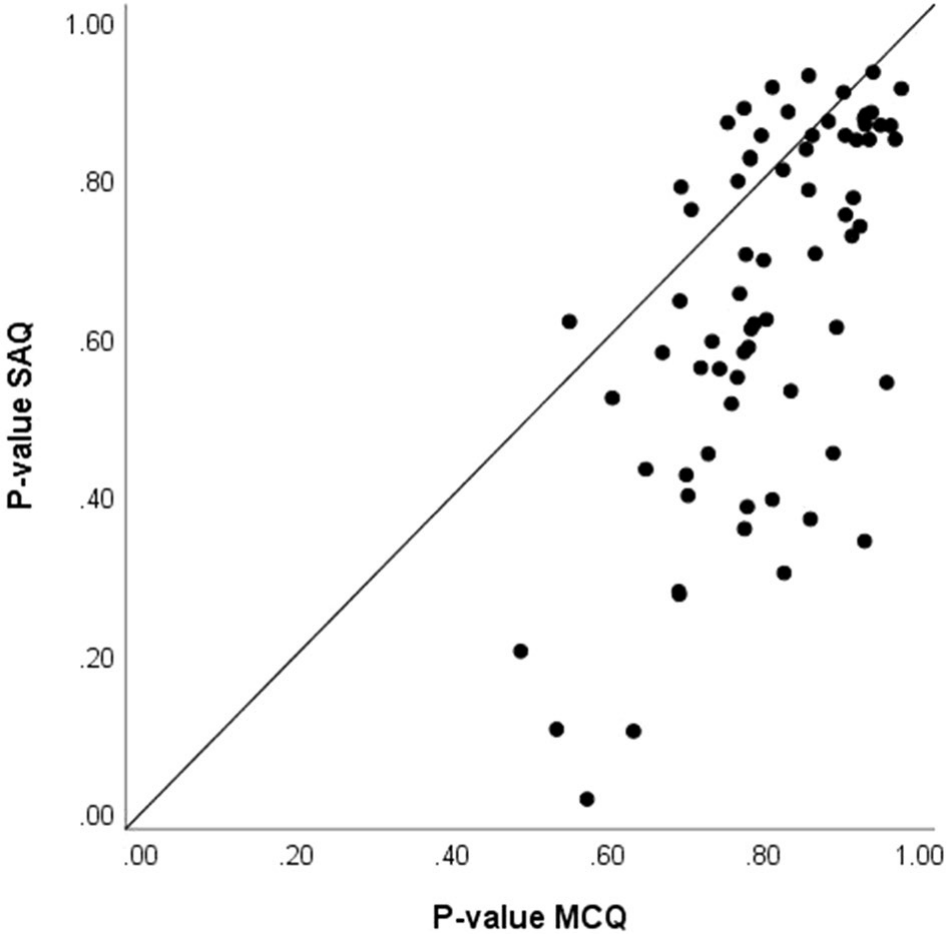
Percent correct (p-values) for 71 items in multiple-choice and short-answer formats

As we have stated, our primary interest is in evaluating the relative difficulty, discrimination, and time requirements for items in the two formats. With regard to difficulty our results show that in the aggregate MCQs were easier than SAQs: the average p-value for the MCQ items is 0.80 whereas for the SAQ items, it is 0.65. To more closely examine these results, we first considered whether the relative difficulty of the items remains the same across formats. To examine this, we produced a scatter plot and correlation coefficient for the p-values for the matched items across formats. Figure 2 presents the scatter plot of the p-values for the 71 scored items. The value for the SAQ format is plotted against the value for the MCQ format for the same question. The correlation between formats is 0.64. For 13 items, the short-answer form is at least modestly easier than the MCQ form; however, the short-answer form is more difficult for most items. Four of the items that were relatively difficult as MCQs became very difficult when presented as SAQs. We examined the individual responses for these four items for evidence that difficulty resulted from ambiguity in the question that might make it inappropriate for use as an SAQ. For three of the items, nothing about the responses pointed in that direction. Many of the incorrect responses to the SAQ version were variations on the distractors from the MCQ form of the item. The fourth item had a lead-in that asked, “What is the most appropriate next step in diagnosis?” The keyed answer was, “No further testing is indicated.” As such, it is not an inherently unfair question, but in the absence of the option list, it is easy to see how examinees might have assumed that some positive diagnostic step was expected—the presence of the options would have prompted examinees to at least consider the possibility that no further testing is needed.

**Fig. 3:**
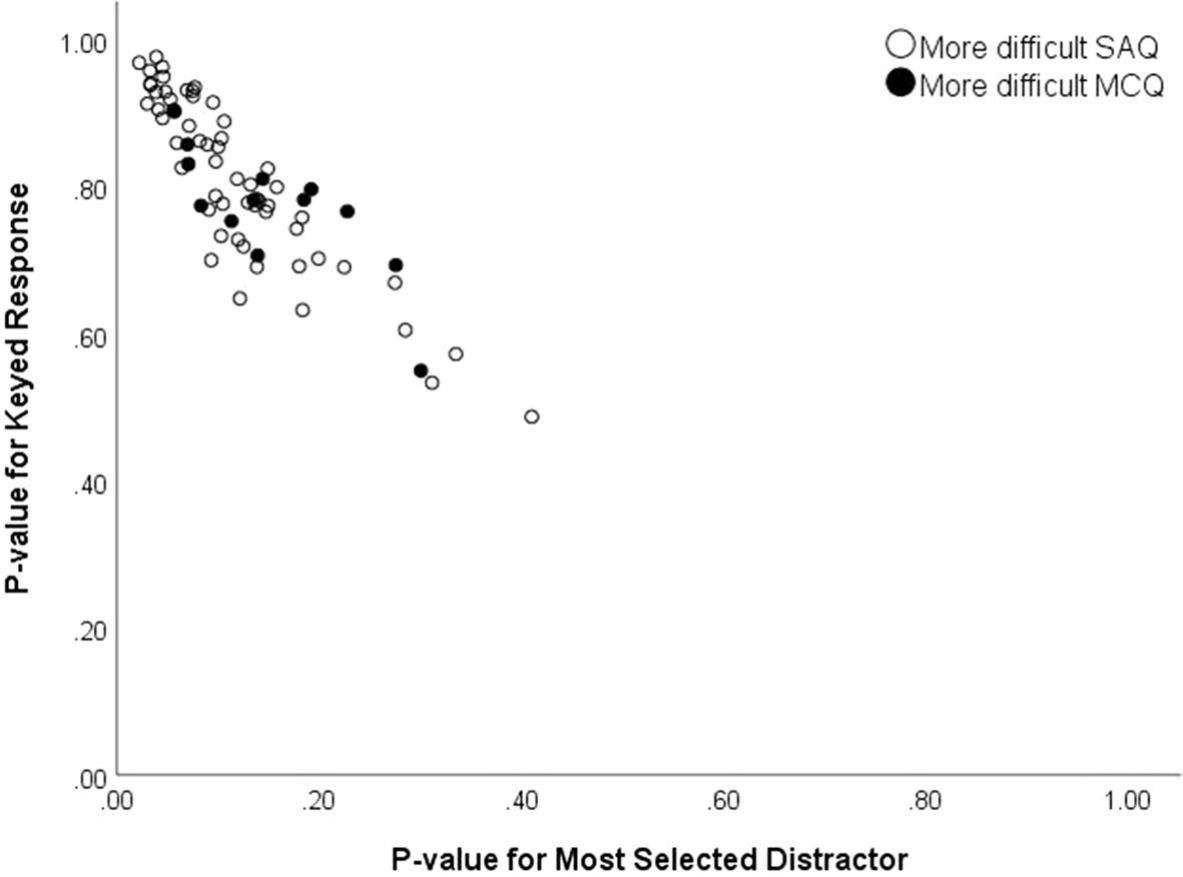
Scatter plot of the proportion of examinees selecting the correct answer and the most frequently selected distractor for items for which the SAQ format was more difficult and items for which the MCQ format was more difficult

The 13 items above the diagonal in Fig. 2 warrant closer examination; these items were easier in the SAQ format. The idea that selecting a response may be more difficult than constructing one is a bit counterintuitive but not surprising. Previous authors have reported this phenomenon—referring to it as *negative cueing* (e.g., Sam et al., 2019; Schuwirth et al., 1996). Previous authors have hypothesized that this performance pattern would be associated with items that have a very attractive distractor. Figure 3 presents a scatter plot of the item difficulties for the 71 MCQs plotted against the proportion of examinees selecting the most attractive distractor for the same item. Different markers are used for the items where the SAQ version was more difficult (empty circle) and the MCQ version was more difficult (filled circle). Although the hypothesis of negative cueing seems reasonable, the figure shows no clear pattern to support the hypothesis. The average p-value for the most selected distractor is not significantly greater for the items for which the MCQ version is more difficult than the SAQ version (t = − 1.418, p = 0.173).

### Discrimination

**Fig. 4:**
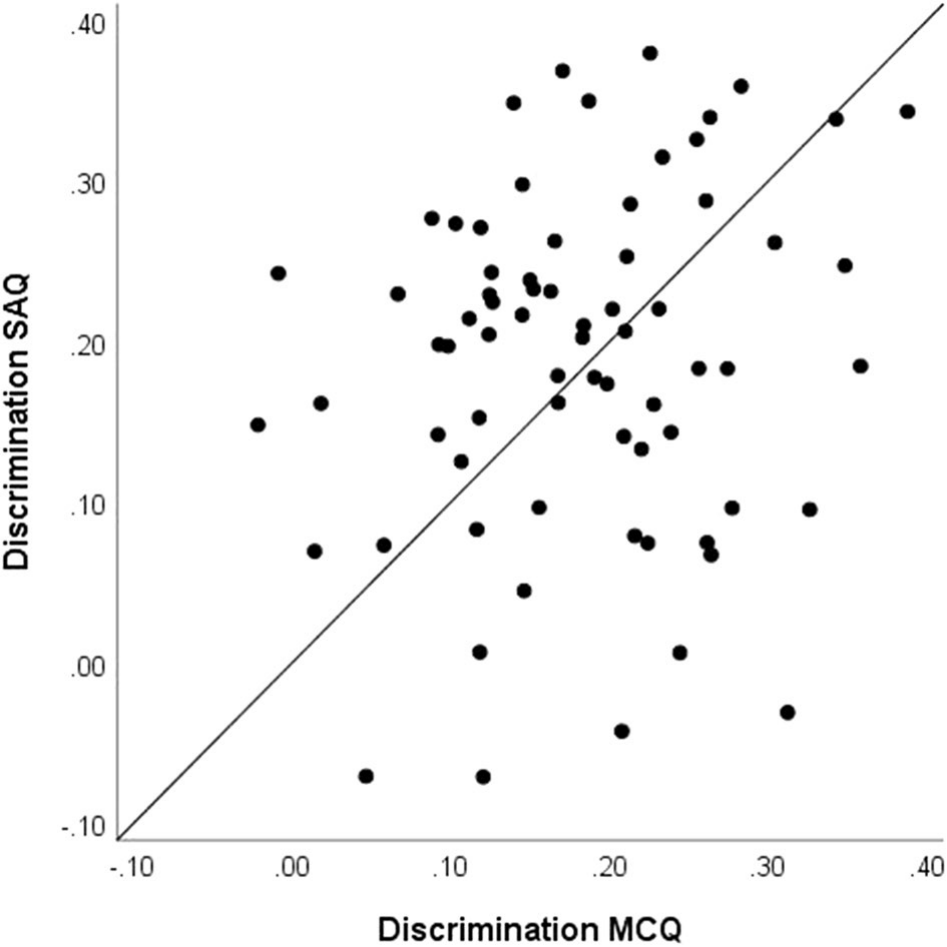
Discrimination values for 71 items in multiple-choice and short-answer formats

Figure 4 presents a scatter plot of the discrimination indices for the two item formats. The results show little, if any, difference between the values for the two formats and the values appear weakly related at most: the mean discrimination is 0.18 for the MCQs and 0.19 for the SAQs; and the observed correlation between the formats is 0.17. This result is interesting when we consider that the discrimination index we are reporting represents the correlation between the individual studied items and the score each examinee achieved on the operational items on the test. That test score is based entirely on MCQs. This suggests that the SAQs correlate with the proficiency measured by MCQs as (or more) strongly as the MCQs correlate with that proficiency. It would appear that the specific content of the stem has little impact on the discrimination across formats, suggesting a strong interaction between stem content and format.

The presence of negative discrimination indices (in both formats) also warrants comment. The studied items used in this research met the requirements for operational use before inclusion in this study. This includes the requirement that they all had positive point-biserial correlations in the context of prior use. For the MCQs, this is essentially the same index used in this study; for the SAQs, it is similar. This suggests that a small number of items will drop below zero due to sampling error. The fact that four items display this pattern for the SAQs and only two for the MCQs (no item fell below zero in both formats) may indicate that the format change impacted the discrimination for some items; the sample is too small to draw conclusions. It may be worth noting that two items with negative discrimination as SAQs were among the four SAQs with very low p-values referenced previously.

### Timing

**Fig. 5:**
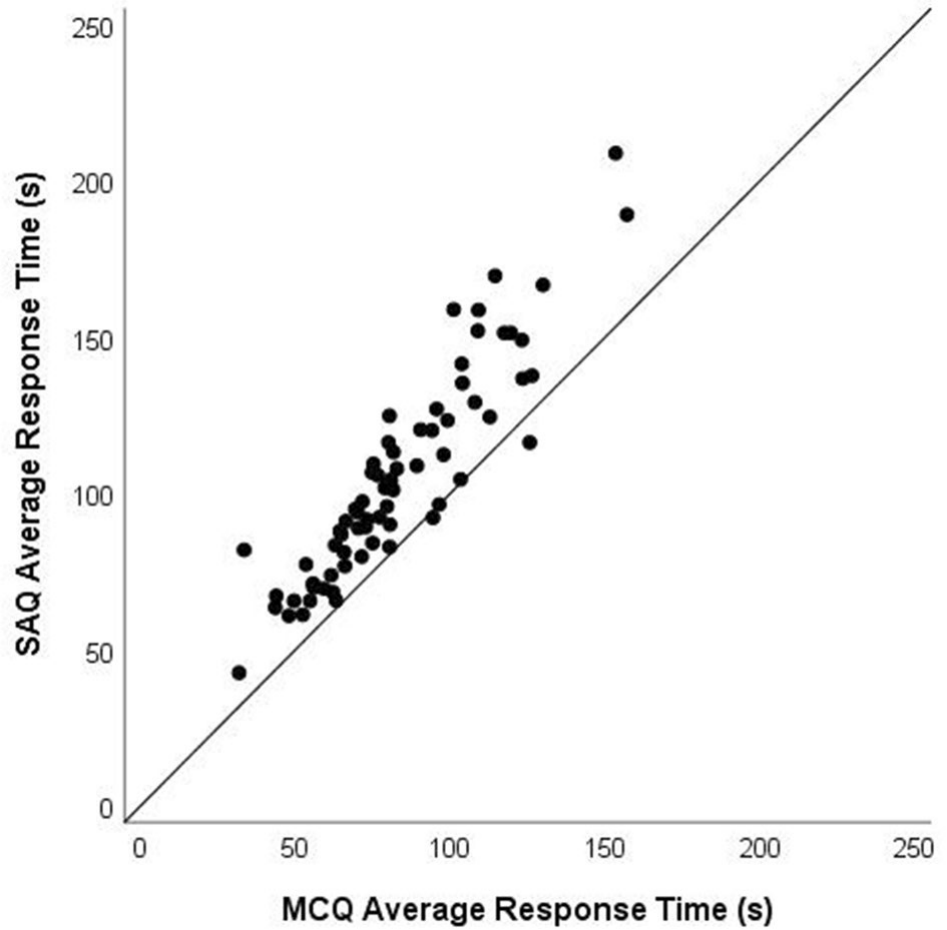
Average response times for 71 items in multiple-choice and short-answer formats

Figure 5 presents a scatter plot of the timing information for the two formats. The results unequivocally show that the SAQ format generally requires more time than the MCQ format. The mean response time for the SAQs was 105 s (SD = 33); for the MCQs, this value was 83 s (SD = 26). On average, the SAQs took 27% longer to complete—approximately 22 additional seconds for each item. The correlation of 0.92 between response time for the two formats and examination of the scatterplot suggest that the relative time requirement remains stable across formats, with the additional time used for SAQs being generally uniform across items. (Standard timing for the test used in this research was 90 s per MCQ item.)

## Discussion

Although the results of the present study are generally in line with those from earlier comparisons of short-answer and multiple-choice items, the present study does provide important information that was not available from previous work. Many of the previous studies: (1) used small samples of examinees (Heemskerk et al., 2008; Newble et al., 1979; Norman et al., 1987); (2) used volunteers who were testing under low-stakes conditions (this is the case even for the studies that used larger samples—i.e., Sam et al. (2018) and Sam et al. (2019)); and (3) the results of many previous studies are based on a design in which the same examinees are asked to respond the same item in both formats so that the effect of item format is confounded by the effect of seeing an given item for a second time. The current study collected data from a large sample of examinees testing under high-stakes conditions and used random assignment. Additionally—and perhaps most importantly—the present study quantifies the difference in response time for the two formats.

### Difficulty

As with most previous studies, our results show that SAQs generally are more difficult than MCQs. Some previous authors have interpreted this as evidence that MCQs are defective as measures of clinical competence (Newble et al., 1979; Sam et al., 2019). Given the high correlations between the scores from the two formats (Norman et al., 1987; Schuwirth et al., 1996), it seems more reasonable to conclude that the two formats measure very similar constructs on somewhat different scales. The very similar point-biserial correlations for the SAQs and MCQs reported in this study further supports the idea that the two formats assess similar if not identical constructs. That said, the potential for examinees to guess, the potential to use test-taking strategies to eliminate incorrect options, and most importantly the fact that MCQs call upon the examinee to recognize rather than recall the correct answer, remain concerns when tests comprise MCQs. There is clearly a need for more research in this area. Think-aloud studies and eye-tracking studies comparing examinee response processes across the two formats have the potential to provide important insights into the extent to which recognition (rather than recall) of the response impacts the construct being assessed.

In the present study, four items became sufficiently difficult when put in the SAQ format that they would likely be excluded from use based on common standards of item eligibility for test development. As previously noted, two of the most difficult SAQs had negative discrimination indices. This is further evidence that the SAQ format may have limitations that warrant more detailed study.

In some circumstances, the fact that the SAQs tend to be more difficult may be beneficial. If a testing program wishes to take advantage of adaptive testing technology to maximize the precision of scores across the proficiency range, having relatively difficult items in the pool will be important. The reverse may also be true. For example, in an application such as the USMLE examinations, the increased difficulty might be viewed as a liability as the difficulty of the current tests is well targeted to the proficiency at the cut score. This maximizes precision where it is most needed: for those examinees near the cut score. If the difficulty of the items were systematically shifted by making the mean p-value lower, the precision of the scores for most of the examinees would be increased at the expense of reducing the precision for those examinees whose pass/fail status is most in question.

It is generally assumed that SAQs are more difficult than MCQs because it is easier to recognize the correct answer than to recall it. Sam et al. (2019) examined the hypothesis that the relative difficulty of the two formats was a function of increased scores resulting from random guessing. This may seem like an appealing explanation, but numerous researchers have argued that truly random guessing is rare (e.g., Baldwin, 2021; Lindquist & Hoover, 2015); examinees typically make use of partial knowledge. Lindquist and Hoover, citing Norton (1950), suggest that one sensible estimate of the maximum proportion of examinees guessing randomly on a given item is the proportion selecting the least popular distractor. If guessing is random, examinees using this strategy will select each option in a similar proportion. In practice it is not uncommon for that proportion to be low. For the items used in this study, the mean proportion of examinees selecting the least commonly chosen distractor was 0.01. The actual difference in difficulty between the two formats is more than an order of magnitude greater than the difference that the presence of random guessing would explain.

### Discrimination

The results reported here are consistent with previous research suggesting that SAQs are similar to MCQs in terms of reliability or discrimination. This supports the view that in situations in which it is important that the examinee generate the response rather than select it, SAQs may offer a reasonable alternative to MCQs.

Explaining why SAQs are generally more difficult than MCQs may be relatively straightforward: generating the correct answer is generally more difficult than selecting it. Additionally, in many cases, the MCQ version of an item may be less ambiguous than the SAQ version. Explaining the general trend in the literature (and the results reported in this paper)—that SAQs may be more discriminating than MCQs—is more difficult. One possible explanation is that the random guessing associated with MCQs introduces noise into the scores, which lowers the discrimination. This may be a small factor, but given the low level of truly random guessing, the impact is likely to be modest.

Another potential explanation is linked to the fact that SAQs are more difficult than MCQs. The information available from a test item is maximized when the item is well-targeted to the examinee—or, more generally, to the population of examinees who take the test. That information is maximized when the examinee has a 0.5 probability of answering correctly.^1^ The more difficult SAQ format may display higher discrimination (and higher reliability) simply because the mean response probability is closer to that point of maximum information. This general relationship is why computer-adaptive tests can provide greater precision with fewer items. It is also the basis for using item response theory models to build tests that have maximum precision at the cut score. In practice, it is impossible to change an item’s difficulty while holding all other characteristics of the item constant. However, this manipulation is possible within the theoretical framework of item response theory. The appendix reports simulation results designed to evaluate the impact of such a change using that framework. The simulation results suggest that the greater discrimination reported for the SAQs in this paper (and in much of the other literature on SAQs) could well be accounted for by the increase in difficulty.

### Timing

As we noted, this may be the first paper to provide detailed interpretable results about the time requirements for responding to items in the two formats. The difference in response time showed that SAQs consistently take longer than MCQs. This is not surprising: it takes longer to type a response than to select one. However, 22 s is substantially longer than it would take a typical medical student to type 60 characters. It appears that the cognitive processing time for this format exceeds that for MCQs. This difference in processing time may have implications for understanding the difference in the cognitive processes used to respond to the two formats. The difference may also have practical implications. The practical importance of this time difference will depend on the context in which the assessment occurs. When available testing time is a limiting factor, using a more time-intensive format may necessitate tests with fewer items. To provide a sense of the potential impact of using more time-intensive SAQs, consider the reliabilities reported by Sam et al. (2019): 0.69 for MCQs and 0.73 for SAQs. Based on the timing results from our study, if the comparison were based not on an equal number of items but on equal testing time, the MCQ test would have 28% more items, and the MCQ reliability would be 0.74.

It is worth noting that although these results are in line with previous research showing that SAQs are more time intensive than MCQs, our results—collected within an experimental design using random assignment—differ by nearly an order of magnitude from those reported by Sam, et al. (2019). In our study the ratio of testing time for SAQs to MCQs was 1.27/1.00; in the Sam, et al. study that ratio was 3.42/1.00. This latter ratio will strongly favor MCQs if testing time is a limited factor in the administration.

### Additional considerations

The results reported here argue for the potential application of SAQs for assessment in the health sciences—including licensure and certification testing. As we noted at the beginning of this paper, automated scoring must be effective and efficient for that application to be practical. The available evidence shows that NLP technology has advanced during recent years and that computer-based scoring for SAQs in large-scale and high-stakes tests is achievable (see, for example, Sam et al., 2019; Suen, et al., 2023; Yamamoto et al., 2017; Yaneva & von Davier, 2023).

Despite our optimism, considerable research is still needed to better understand SAQs. The MCQ format has been carefully studied for more than a century. The format has been used in physician licensure assessment for nearly 70 years (Hubbard & Levit, 1985). Our knowledge of how to write high-quality SAQs needs to catch up to our ability to write MCQs. In the present study, 29 of the original 100 studied items were set aside as inappropriate for scoring after collecting initial responses. Six additional items produced statistical characteristics that would have removed them from use in operational testing. These results occurred with item content that had already been pretested, carefully vetted, and accepted for use in MCQ form on the USMLE Step 2 CK examination.

Developing a scoring key may be problematic even when the items function properly. The inter-judge agreement for the 71 items used in this study was high; it was not perfect. It is possible that with more experience in developing SAQs for high-stakes testing, improvements will be made in developing scoring rules or writing items that produce responses that can be scored with simpler rubrics. Given that the quality of the scores produced by an automated system will be significantly limited by the quality of the human scores the system intends to replace, this limitation still warrants consideration.

An important concern with MCQ items cited in the literature is that test-taking strategies rather than clinical knowledge may allow examinees to answer correctly (e.g., Newble et al., 1979; Sam et al., 2016). With professionally developed test material, considerable effort is made to remove such cues as part of careful editorial procedures. Unfortunately, the problem of writing items that are both unambiguous and provide no unintended cuing does not go away when we use SAQs. However, the problem with SAQs is likely the opposite of that typically associated with MCQs. To respond correctly to SAQs, examinees must have the required clinical knowledge and correctly interpret the item writers’ intentions. Even an apparently constrained question like, “What is the most likely diagnosis?” requires the examinee to decide on the appropriate specificity level. Less constrained questions relating to the next steps in diagnosis or treatment require even more interpretation.

## Conclusions

In general, it is reasonable to view the results of this study as supporting the potential use of SAQs. With wider use and more study, our collective knowledge of how to write effective SAQs will likely increase. At the same time, we will probably gain important insight into when SAQs will improve assessment. There are some content areas where it will be difficult to write effective SAQs; the same is true for MCQs. It may be that the two formats (at least partially) complement each other in this respect. It is also clear that there are some assessment contexts where the relative difficulty of SAQs will be an advantage; in different contexts, this same characteristic will be a liability. The longer response time for SAQs is a clear disadvantage, but other benefits may compensate for this limitation. Based on our literature review and the results of this study, both MCQs and SAQs have advantages. As the potential to machine score SAQs becomes a reality, the challenge for test developers will be how best to leverage those advantages to improve assessment.

## Figures and Tables

**Fig. 6:**
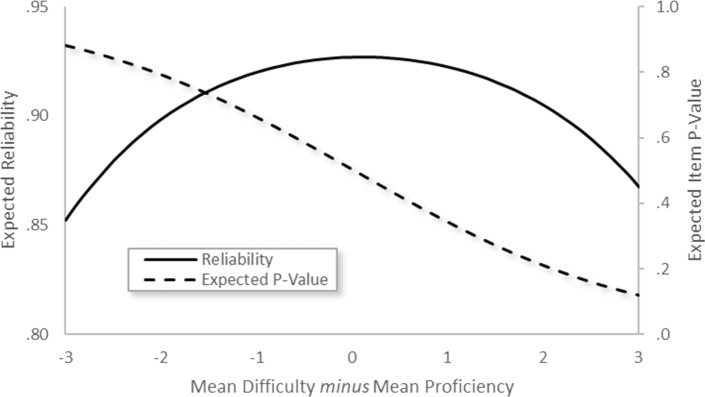
Expected Reliability and P-Value. Note: Expected reliability (KR 20; scale shown on the left axis) and expected p-value (scale shown on right axis) as a function of the difference between mean item difficulty and mean simulee proficiency. Reliability is greatest when mean item difficulty and mean examinee proficiency are equal (p-value) $\approx 0.50$

## Data Availability

The test materials and examinee responses that are the basis for this research are secure materials and cannot be publicly disclosed.

## The relationship between item difficulty and reliability

Earlier in the paper, we discussed the fact that changing the difficulty of a set of items could change reliability or discrimination for those items. It is, of course, impossible to change the difficulty of an item without—at least potentially—impacting discrimination. We can, however, examine the relationship between the difficulty and reliability for a set of items and a given examinee sample using a theoretical model that relates examinee proficiency to the probability of a correct response. Item response theory models describe the probability of observing a correct response on an item (conditioned on examinee proficiency) as a function of one or more item parameters. For example, the 2-parameter logistic item response theory model describes the probability of a correct response as a function of examinee proficiency, item difficulty, and item discrimination:

A1 $$P_{i} (\theta |a_{i} ,b_{i} ) = \frac{1}{{1 + e^{{ - a_{i} (\theta - b_{i} )}} }},$$

where *a*_*i*_ and $b_{i}$ are the item discrimination and difficulty parameters, respectively, for item $i$; $\theta$ is the examinee proficiency parameter; and $P_{i} (\theta |a_{i} ,b_{i} )$ is the probability of a correct response conditional on $\theta$ for a given item $i$ described by the parameters $a_{i}$ and $b_{i}$. Using this model, it is straightforward to simulate examinee responses after specifying the distribution of examinee proficiency and the item parameters.

We simulated 100 items by sampling item difficulties from a standard normal distribution and item discriminations from a uniform distribution [0.4, 1.4]. Likewise, 1000 examinees were simulated by sampling examinee proficiencies also from a standard normal distribution. Item difficulties then were manipulated by adding a constant, which was varied as a study condition from -3 to + 3 in increments of 0.1. For each constant and each of 1000 replications, model-based response data were simulated using the model given in Eq. (A1) and the sampled item and examinee parameters. For each condition and replication, item difficulty was summarized by calculating the mean item p-value (proportion correct) and reliability was calculated using the well-known KR 20 formula. (For the data used in this research and this simulation, KR 20 is identical to coefficient alpha.)

Figure 6 shows the expected item p-value and reliability as a function of the difference between mean proficiency and mean difficulty. (Note that the scale for the reliability results is shown on the left axis, and the scale for the p-value results is shown on the right axis.) Reliability is maximized when items have a p-value of (approximately) 0.5. In this way, it follows that if a set of MCQ items were relatively easy for a given population (i.e., with an expected p-value > 0.5), replacing these items with more difficult short answer versions would increase reliability even if everything else about the items remained unchanged.

We also replicated this simulation study by adjusting the items to have an expected p-value of 0.80 and an expected point biserial of 0.18, which corresponds to the empirical values we reported for MCQs in this study. The point biserial increased when these items were adjusted to have a p-value of 0.65 (the value we observed for the SAQs). The results of both simulations consistently show that a change in item difficulty of this magnitude could produce a change in point-biserials similar to the differences in discrimination (or reliability) reported in SAQ/MCQ comparisons.

## Footnotes

1 When “guessing” is substantial, the point of maximum information will shift upward modestly.
